# *MUTYH* gene variants and breast cancer in a Dutch case–control study

**DOI:** 10.1007/s10549-012-1965-0

**Published:** 2012-02-02

**Authors:** Astrid A. Out, Marijke Wasielewski, Petra E. A. Huijts, Ivonne J. H. M. van Minderhout, Jeanine J. Houwing-Duistermaat, Carli M. J. Tops, Maartje Nielsen, Caroline Seynaeve, Juul T. Wijnen, Martijn H. Breuning, Christi J. van Asperen, Mieke Schutte, Frederik J. Hes, Peter Devilee

**Affiliations:** 1 Department of Clinical Genetics, Leiden University Medical Center, Leiden, The Netherlands; 2 Department of Human Genetics, Leiden University Medical Center, P.O. Box 9600, 2300 RC Leiden, The Netherlands; 3 Department of Pathology, Leiden University Medical Center, Leiden, The Netherlands; 4 Department of Medical Statistics, Leiden University Medical Center, Leiden, The Netherlands; 5 Department of Medical Oncology, Josephine Nefkens Institute, Erasmus University Medical Centre, Rotterdam, The Netherlands; 6 Department of Medical Oncology, Erasmus MC–Daniel den Hoed Cancer Center, Rotterdam, The Netherlands; 7 Present Address: Department of Pathology, VU University Medical Center, Amsterdam, The Netherlands; 8 Present Address: Department of Genetics, University Medical Center Groningen, Groningen, The Netherlands; 9 Present Address: Lorentz Center, Leiden, The Netherlands

**Keywords:** MUTYH, Breast cancer, BRCAx, Case–control study, Genotyping

## Abstract

The *MUTYH* gene is involved in base excision repair. *MUTYH* mutations predispose to recessively inherited colorectal polyposis and cancer. Here, we evaluate an association with breast cancer (BC), following up our previous finding of an elevated BC frequency among Dutch bi-allelic *MUTYH* mutation carriers. A case–control study was performed comparing 1,469 incident BC patients (ORIGO cohort), 471 individuals displaying features suggesting a genetic predisposition for BC, but without a detectable *BRCA1* or *BRCA2* mutation (BRCAx cohort), and 1,666 controls. First, for 303 consecutive patients diagnosed before age 55 years and/or with multiple primary breast tumors, the *MUTYH* coding region and flanking introns were sequenced. The remaining subjects were genotyped for five coding variants, p.Tyr179Cys, p.Arg309Cys, p.Gly396Asp, p.Pro405Leu, and p.Ser515Phe, and four tagging SNPs, c.37-2487G>T, p.Val22Met, c.504+35G>A, and p.Gln338His. No bi-allelic pathogenic *MUTYH* mutations were identified. The pathogenic variant p.Gly396Asp and the variant of uncertain significance p.Arg309Cys occurred twice as frequently in BRCAx subjects as compared to incident BC patients and controls (*p* = 0.13 and *p* = 0.15, respectively). The likely benign variant p.Val22Met occurred less frequently in patients from the incident BC (*p* = 0.03) and BRCAx groups (*p* = 0.11), respectively, as compared to the controls. Minor allele genotypes of several *MUTYH* variants showed trends towards association with lobular BC histology. This extensive case–control study could not confirm previously reported associations of *MUTYH* variants with BC, although it was too small to exclude subtle effects on BC susceptibility.

**Electronic supplementary material:**

The online version of this article (doi:10.1007/s10549-012-1965-0) contains supplementary material, which is available to authorized users.

## Introduction

The *MUTYH* gene (muty homolog [*Escherichia coli*]*,* MIM *604933) encodes a DNA glycosylase involved in base excision repair (BER). MUTYH, in cooperation with OGG1 and NUDT1, prevents G:C > T:A transversions, resulting from 8-oxo-G:A mispairs generated by oxidative damage. *MUTYH* germline mutations cause the recessively inherited phenotype: *MUTYH*-associated polyposis (MAP, MIM #608456), via somatic G:C > T:A mutations in *APC* and *KRAS* [1–3]. The mono-allelic (heterozygous) pathogenic *MUTYH* mutation frequency is 1–2% in the population of European descent. Bi-allelic mutations (homozygous or compound heterozygous) are estimated to occur in 1:2,500–1:10,000. In Dutch MAP patients, three founder mutations (p.Tyr179Cys, p.Gly396Asp, and p.Pro405Leu) account for up to 90% of the pathogenic *MUTYH* mutations [4]. Bi-allelic *MUTYH* mutation carriers have a 28-fold increased colorectal cancer (CRC) risk, while mono-allelic carriers have a moderately increased CRC risk (OR up to 1.34) [5].

Recently, two studies suggested a possible breast cancer (BC) risk for bi-allelic *MUTYH* mutation carriers. A significantly increased incidence (4/22, 18%) of BC was found among Dutch female bi-allelic *MUTYH* mutation carriers, as compared to the Dutch population [4]. Of these four BC patients, three were diagnosed before the age of 55 years and three had multiple breast tumors. In a next study, combining Dutch, German, and British MAP patients, 8/118 (6.8%) females and 1/158 (0.6%) males were diagnosed with BC. The standardized incidence ratio (SIR) was significantly increased for male BC and also for female BC, when taking into account multiple breast tumors per patient [6]. Furthermore, heterozygous *MUTYH* founder mutations were found to be significantly increased among index-patients with BC and/or CRC, from families with both BC and CRC (6/138, 4.3%), as compared to controls (23/1192, 1.9%), with exclusion of families with colorectal polyps [7], indicating a possible BC risk for *MUTYH* mutation heterozygotes.

Several independent findings support a possible role of *MUTYH* in BC. First, like *MUTYH*, many BC genes encode proteins involved in the DNA damage response, e.g., *BRCA1/2* and *CHEK2*. Second, oxidative DNA damage due to hormonal metabolism, including 8-oxoG, is believed to contribute to BC [8–12]. Third, somatic *APC* mutations have been described in a substantial proportion of breast carcinomas (13/70, 18%). Of the detected mutations, 5/15 were substitutions, of which 4 (80%) were G:C > T:A [13]. Fourth, an increased frequency of mammary tumors was found in both *MUTYH*- and *APC*-deficient mice compared to mice with an *APC* deficiency only [14]. Fifth, besides nuclear DNA damage, mitochondrial DNA (mtDNA) damage probably contributes to BC and *MUTYH* is abundantly expressed in mitochondria [15]. Finally, variants in the DNA mismatch repair (MMR) genes have been found to be weakly associated with BC [16].

The aim of this study was to extensively validate an association of *MUTYH* mutations with BC, as suggested in three previous studies [4, 6, 7]. We first explored the *MUTYH* mutation spectrum in BC patients by sequencing the entire *MUTYH* coding region in 303 cases not selected for family history. We hypothesized that the *MUTYH* mutation spectrum could differ between BC and MAP patients. Next, we established the frequency of pathogenic *MUTYH* founder mutations in 1,469 incident BC patients and 1,666 controls. We also explored whether common *MUTYH* variants and haplotypes were associated with BC, by genotyping tagging single nucleotide polymorphisms (SNP). Finally, we investigated if non-BRCA1/BRCA2 familial BC patients (called BRCAx) have an increased *MUTYH* variant frequency, as compared to incident BC patients and controls. BRCAx patients might harbor so far undetermined high-risk gene variants and/or variants in multiple interacting high and low risk genes [17, 18]. This study reports the largest series of incident BC patients as reported to date and for the first time *MUTYH* mutation frequencies in BRCAx patients.

## Patients and methods

### Patient samples

Two BC patient groups were studied. The first group consisted of 1,518 incident BC patients, sampled for DNA examination in Rotterdam and Leiden between 1997 and 2008 (“ORIGO” cohort) [19]. Of this cohort, 1,469 remained after exclusion of patients with known *BRCA1* (33), *BRCA2* (12), *MLH1* (2), *MSH2* (1), and *PTEN* (1) mutations. The second group was designated the “BRCAx” cohort, consisting of 471 unrelated subjects who underwent *BRCA1/2* mutation screening at the Leiden University Medical Center (LUMC), The Netherlands, based on their family history and/or age at cancer diagnosis, but were not identified as BRCA1/2 mutation carriers. The 471 subjects included 376 BC patients (of whom 10 males), 29 ovarian cancer patients, and 7 patients with both breast and ovarian cancer. The remaining 59 subjects were tested for *BRCA1/2* mutations because of a strong family history of breast, ovarian or other *BRCA1/2*-associated tumors [20]. As controls, 1,666 individuals were studied, originating from the South-Western part of the Netherlands, consisting of 165 partners of members from BC families, 254 female subjects screened for non-cancer-related genetic diseases and 773 healthy female blood donors [7, 19]. Approval from the Medical Ethical Review Board of Leiden University Medical Centre and informed consent from all the individuals had been obtained (P06.060).

### Sequencing and genotyping

Molecular genetic tests were performed on blood-derived DNA. Direct sequencing was performed for the coding region and exon/intron boundaries of *MUTYH* as described [4, 21]. Variants were annotated using NM_001128425.1 as reference sequence (www.lovd.nl/MUTYH, www.ncbi.nlm.nih.gov/genbank/) [22]. Genotyping of c.536A>G (p.Tyr179Cys), c.925C>T (p.Arg309Cys), c.1187G>A (p.Gly396Asp), c.1214C>T (p.Pro405Leu), and c.1544C>T (p.Ser515Phe) was done using Taqman^®^ assays on an ABI 7500 instrument (Applied Biosystems, Inc., Foster City, CA, USA). The variants c.37-2487G>T, c.64G>A (p.Val22Met), c.504+35G>A, and c.1014G>C (p.Gln338His) were typed by Sequenom iPlex^®^ (Sequenom, Inc., San Diego, CA, USA). Of the incident BC group, 214 and 291, of the 303 sequenced samples, were also analyzed by Taqman and Sequenom genotyping, respectively, with 100% concordancy of results. Primer sequences, PCR, Taqman, Sequenom, and sequence conditions are available on request. *In silico* analysis of potential deleterious effects of new *MUTYH* variants was performed with Alamut^®^ Software (Interactive Biosoftware, Rouen, France).

### Statistics

Chi-square tests were performed to test for differences in genotype frequencies between the study groups and the binary logistic regression analyses were used to estimate odds ratios, with the SPSS package version 16.0 (SPSS, Inc., Chicago, IL, USA). Associations of different phenotypical parameters with genotypes were analyzed by Chi-square tests, one-way analysis of variance (ANOVA), and binary logistic regression. Where applicable, *p* values were calculated by a Fisher’s exact test or estimated by a Monte–Carlo simulation. A *p* value below 0.05 was considered as statistically significant.

Power calculations showed that with 1,300 incident patients and 1,300 controls (a rounded estimate taking into account missing genotypes), and a heterozygote frequency of 2% in the controls, we had 80% power to detect heterozygote frequencies above 4% in the patients (OR 2.0) at *p* < 0.05. With 450 BRCAx patients and 1,300 controls there is a power of 80% to detect a heterozygote frequency of 5% or higher (OR 2.6).

Unphased genotypes of the nine variants were loaded into Haploview software version 4.1 to test for associations of haplotypes and to test for Hardy–Weinberg equilibrium (HWE) [23].

## Results and discussion

### Sequencing of the *MUTYH* coding region

We first evaluated the spectrum of *MUTYH* variants by direct sequencing of the entire *MUTYH* coding region in 303 consecutively collected DNA samples from incident BC patients (ORIGO cohort). To enrich genetically susceptible cases, we selected cases with either a diagnosis before 55 years of age (*N* = 247) or with multiple primary BCs (*N* = 56, Table 1). Detected variants were compared to data from literature (www.lovd.nl/MUTYH, [1, 22, 24, 25] and dbSNP (build 133, www.ncbi.nlm.nih.gov/projects/SNP). In the 303 patients, no bi-allelic pathogenic mutations were found. Of the three Dutch pathogenic founder mutations, only p.Gly396Asp was detected, in two heterozygous patients (0.7%). The mutations, p.Tyr179Cys and p.Pro405Leu, were not found or any other known pathogenic mutations. This was lower compared to the total prevalence of heterozygous pathogenic *MUTYH* mutations in control populations of European descent (1–2%) [26].

**Table 1:** *MUTYH* variants detected by direct sequencing in 303 incident BC patients diagnosed before the age of 55 years, of whom 56 had multiple primary breast tumors

Variant^a^	*N* = 303^c^		*N* = 56 with >1 breast tumor^c^		Classification^d^	Rs number^d^
	No. het + hom	% MAF	No. het + hom	% MAF		
c.-127C>T	20	3.3	4	3.6	Polymorphism	rs3219466
c.36+75C>G	2	0.3	0	0	VUS	rs3219467
c.56G>A (p.Arg19Gln)	1	0.2	0	0	VUS, new	–
c.64G>A (p.Val22Met)	29	4.8	2	1.8	Polymorphism	rs3219484
c.157+30A>G	29	4.8	4	3.6	Polymorphism	rs3219485
c.312C>T (p.=)	1	0.2	0	0	VUS	–
c.388+56G>A	2	0.3	0	0	VUS	–
c.504 +35G>A^b^	58 + 5	11.2	13 + 1	13.4	Polymorphism	rs3219487
c.690+21C>A	4	0.7	0	0	VUS	–
c.925C>T (p.Arg309Cys)	3	0.5	0	0	VUS	–
c.998-27G>A	3	0.5	0	0	VUS	–
c.1014G>C (p.Gln338His)	103 + 15	21.9	20 + 4	25.0	Polymorphism	rs3219489
c.1186+46G>A	1	0.2	1	0.9	VUS, new	–
c.1187-27C>T	6	1.0	0	0	Polymorphism	rs3219490
c.1187G>A (p.Gly396Asp)	2	0.3	0	0	Pathogenic	rs36053993
c.1477-40G>C^b^	58 + 5	11.2	13 + 1	13.4	Polymorphism	rs3219493
c.1518+73C>T	0 + 1	0.3	0	0	Polymorphism	rs3219495
c.1518+90A>G	1	0.2	1	0.9	VUS, new	–
c.1544C>T (p.Ser515Phe)	10	1.7	5	4.5	Polymorphism	–
Total pathogenic	2	0.3	0	0		
Total intronic VUS	13	2.1	2	1.8		
Total coding VUS	5	0.8	0	0		

*het* heterozygous, *hom* homozygous, *MAF* minor allele frequency

^a^No combinations of bi-allelic pathogenic mutations were found. Many patients carried multiple variants, of which the variants found in combination with variants found in 3/303 patients or less are mentioned here: c.56G>A and c.64G>A, c.312C>T and c.1544C>T, c.36+75C>G and c.-127C>T (2/2 carriers), c.388+56G>A and c.-127C>T (1/2), c.925C>T and c.504 +35G>A and c.1477-40G>C (1/3), c.998-27G>A and c.504 +35G>A and c.1477-40G>C (1/3), c.1186+46G>A and c.-127C>T and c.1014G>C

^b^Minor alleles of these two variants were detected in the same patients, indicating strong LD

^c^The 303 consecutively accrued incident BC patients selected from the ORIGO cohort for BC diagnosis before the age of 55 years, among whom 56 had multiple primary breast tumors (bilateral or ipsilateral). This group was slightly enriched for patients with multiple breast tumors, by selection of the last 10 patients for this characteristic

^d^A likely classification based on data and MAF from literature and dbSNP. Two dbSNP variants were also genotyped in this study, but were not detected by sequencing in these 303 samples, namely c.536A>G (not present in these 303 samples, rs34612342) and c.37-2487G>T (outside the sequenced region, rs3219476). The genotyped variant c.1214C>T, p.Arg309Cys, was not detected in the 303 samples and also not present in dbSNP

Eighteen patients (5.9%) carried a variant of uncertain significance (VUS), of which 13 carried an intronic variant (without predicted RNA splice effect). We found three different coding VUS in five patients (c.56G>A (p.Arg19Gln), c.312C>T (p.Tyr104Tyr), and three times c.925C>T (p.Arg309Cys)). The variant, p.Arg309Cys, has been described as a VUS in polyposis patients [6, 27]. Its effect was tested in one functional study, and showed normal glycosylase activity [28]. Three of the observed VUS were novel (c.56G>A (p.Arg19Gln), c.1186+46G>A, and c.1518+90A>G), but *in silico* analyses showed no clear indications for pathogenicity (i.e., low Grantham scores, weak nucleotide and amino acid conservation and no predicted effects on RNA splicing were observed using Alamut). Common polymorphisms were found in similar frequencies as in populations of European descent. In the 56 patients with multiple primary breast tumors, the polymorphism c.64G>A (p.Val22Met) was found about half as frequently, and the polymorphism c.1544C>T (p.Ser515Phe) about twice as frequently, compared to the patients with a single breast tumor (Table 1).

### Case–control study by genotyping selected variants

We selected five candidate variants and four tagging SNPs in *MUTYH* for further genotyping in the entire group, totaling the 1,469 incident BC patients (ORIGO cohort), 1,666 controls, and 471 subjects at increased risk for carrying *BRCA1/2* mutations (BRCAx). The candidate variants included the three common Dutch founder mutations (p.Tyr179Cys, p.Gly396Asp, and p.Pro405Leu), and two non-synonymous variants detected more than once in the 303 sequenced patients [p.Arg309Cys (VUS) and p.Ser515Phe (rare polymorphism)]. The four tagging SNPs were selected from the HapMap database (http://hapmap.ncbi.nlm.nih.gov/), as representative for *MUTYH* polymorphisms with minor allele frequencies (MAF) of 5% or higher. Of these four tagging SNPs, three were in the area covered by direct sequencing (p.Val22Met, c.504+35G>A, and p.Gln338His) and one was located outside this area, in intron 1 (c.37-2487G>T) (Table 1). No deviations from HWE were observed in any group.

In the incident BC patient, BRCAx and control groups, no bi-allelic combinations of pathogenic mutations were detected. One control sample was homozygous (bi-allelic) for the rare polymorphism, p.Ser515Phe. Heterozygotes for the polymorphism, p.Val22Met, occurred less frequently among the incident BC patients (9.4%, OR 0.74, 95% confidence interval (CI) 0.59–0.93, *p* = 0.03), as compared to the controls (12.3%), suggesting a protective effect of the minor allele. A similar trend observed in the BRCAx group (Table 2). The frequency of the pathogenic variant, p.Gly396Asp, was approximately twice as high in the BRCAx subjects (2.0%, OR 2.11, 95% CI 0.88–5.03, *p* = 0.13) as in the controls (0.9%), and the incident BC patients (1.0%). Likewise, the frequency of the VUS p.Arg309Cys was twice as high in the BRCAx subjects (1.5%, OR 2.19, 95% CI 0.81–5.89, *p* = 0.15) as in the controls (0.7%), and the incident BC patients (0.4%, Table 2). The increased frequencies of p.Gly396Asp and p.Arg309Cys among BRCAx subjects might suggest an enrichment of *MUTYH* variants in this familial group, analogous to what was found for *CHEK2* [18].

**Table 2:** Genotyping results of nine *MUTYH* variants in patients and controls

Variant/group	Genotype	Controls (1,666)	ORIGO (1,469)			BRCAx (*N* = 471)		
		No. (%)	No. (%)	OR (95% CI)	*p*	No. (%)	OR (95% CI)	*p*
Rare variants		*N* = 1,270	*N* = 1379			*N* = 455		
c.536A>G (p.Tyr179Cys)	AA	1,255 (99.1)	1,363 (99.2)		0.84	452 (99.3)		0.77
	AG	12 (0.9)	11 (0.8)	0.84 (0.37–1.92)		3 (0.7)	0.69 (0.20–2.47)	
c.925C>T (p.Arg309Cys)	CC	1,254 (99.3)	1,364 (99.6)		0.44	447 (98.5)		0.15
	CT	9 (0.7)	6 (0.4)	0.61 (0.22–1.73)		7 (1.5)	2.19 (0.81–5.89)	
c.1187G>A (p.Gly396Asp)	GG	1,252 (99.1)	1,361 (99.0)		1.00	446 (98.0)		0.13
	GA	12 (0.9)	14 (1.0)	1.07 (0.50–2.33)		9 (2.0)	2.11 (0.88–5.03)	
c.1214C>T (p.Pro405Leu)	CC	1,264 (99.9)	1,374 (99.9)		1.00	455 (100)		1.00
	CT	1 (0.1)	2 (0.1)	1.84 (0.17–20.32)		–	–	
Four variants with MAF<1% combined^a^	Major	1,220 (97.3)	1,328 (97.6)		0.71	435 (95.8)		0.15
	Minor	34 (2.7)	33 (2.4)	0.89 (0.55–1.45)		19 (4.2)	1.57 (0.89–2.78)	
c.1544C>T (p.Ser515Phe)	CC	1,212 (96.1)	1,331 (96.9)		0.30	442 (97.1)		0.33
	CT	48 (3.8)	43 (3.1)	0.82 (0.54–1.24)		13 (2.9)	0.74 (0.40–1.38)	
	TT	1 (0.1)	–	–		–		

Variant/group	Genotype	Controls (1,666)	ORIGO (1,469)			BRCAx (*N* = 471)		
		No. (%)	No.	OR (95% CI)	*p*	No. (%)	OR (95% CI)	*p*
Common (tagging) variants		*N* = 1,661	*N* = 1,463			*N* = 471		
c.37-2487T>G^b^	GG	762 (46.8)	636 (45.3)		0.36	207 (44.7)		0.72
	GT	690 (42.4)	607 (43.2)	1.05 (0.91–1.23)		210 (45.4)	1.12 (0.90–1.39)	
	TT	175 (10.8)	161 (11.5)	1.10 (0.87–1.40)		46 (9.9)	0.97 (0.68–1.39)	
c.64G>A (p.Val22Met)	GG	1,429 (87.3)	1,297 (90.1)		0.03	416 (89.8)		0.11
	GA	202 (12.3)	136 (9.4)	*0.74 (0.59–0.93)*		47 (10.2)	0.80 (0.57–1.12)	
	AA	6 (0.4)	7 (0.5)	1.29 (0.43–3.84)		–	–	
c.504+35G>A	GG	1,373 (84.4)	1,181 (82.5)		0.15	377 (82.3)		0.30
	GA	240 (14.8)	236 (16.5)	1.14 (0.94–1.39)		76 (16.6)	1.15 (0.87–1.53)	
	AA	14 (0.9)	15 (1.0)	1.25 (0.60–2.59)		5 (1.1)	1.30 (0.47–3.63)	
c.1014G>C (p.Gln338His)	GG	955 (58.7)	847 (59.9)		0.70	275 (59.9)		0.72
	GC	587 (36.1)	489 (34.6)	0.94 (0.81–1.09)		160 (34.9)	0.95 (0.76–1.18)	
	CC	85 (5.2)	78 (5.5)	1.04 (0.75–1.43)		24 (5.2)	0.98 (0.61–1.57)	
Haplotypes of 5 variants^c^								
1 (All major alleles)	GGGGC	1,554 (61.5)	1686 (61.7)		0.88	562 (61.8)		0.86
2 (37–2487**T**, 1014**C)**	**T**GG**C**C	547 (21.6)	586 (21.4)		0.88	197 (21.7)		0.98
3 (37–2487**T**, 504 + 35**A**)	**T**G**A**GC	209 (8.3)	261 (9.5)		0.11	86 (9.4)		0.30
4 (64A)	G**A**GGC	160 (6.3)	144 (5.2)		0.10	47 (5.1)		0.20
5 (37–2487**T,** 1014**C**, 1544**T)**	**T**GG**CT**	50 (2.0)	42 (1.5)		0.22	13 (1.4)		0.28

Each patient group was compared to the controls. *p* Values for differences between groups were calculated by Chi-square, linear by linear association with Monte–Carlo correction for 2 × 3 tables or exact correction for 2 × 2 tables (2-sided). Odds ratios (OR) with 95% CIs were calculated by binary logistic regression. *p* values below 0.05 and odds ratios with CIs not including the value 1 are italicized

^a^Analysis of the sum of the allelic burden for four rare variants (minor allele frequency <1%) combined, to increase statistical power

^b^In most populations in dbSNP, T is the major allele. However, in European populations, G is the major allele

^c^The five haplotypes from analysis in Haploview, with a frequency above 1%, in order of frequency. Percentages of haplotypes are shown relative to all others. These haplotypes contain major and minor alleles of five variants. Five bases, one for each variant, are shown in the order of location of the variants, from left to right, representing c.37-2487T>G, c.64G>A, c.504+35G>A, c.1014G>C, and c.1544C>T. Haplotype 1 (GGGGC) contains the major alleles of all the five variants. Haplotype 2 (TGGCC) contains the minor alleles of c.37-2487T>G and c.1014G>C. Haplotype 3 (TGAGC) contains the minor alleles of c.37-2487T>G and c.504+35G>A. Haplotype 4 (GAGGC) contains the minor allele of c.64G>A. Haplotype 5 (TGGCT) contains the minor alleles of c.37-2487T>G, c.1014G>C, and c.1544C>T

On the whole, our study showed no significantly increased BC risk associated with *MUTYH* variants. However, for two variants, p.Gly396Asp and p.Arg309Cys, a doubled frequency of heterozygous carriers was found in the BRCAx group, compared to the incident BC group and controls, resulting in a non-significant OR of 2.1–2.2 (Table 3). Interestingly, in a study among Sephardi Jews of North African descent (389 cases and 541 controls), p.Gly396Asp heterozygotes were found to be significantly increased in BC patients (6.7%) compared to controls (3.7%) [29]. However, no significant difference was found for the p.Tyr179Cys and p.Gly396Asp mutations in a study of 691 incident BC patients and 812 controls from Canada [30]. Also, in a large case–control study in subjects from USA and Poland, no major role in BC was found for eight polymorphisms in six BER pathway genes, among which one variant in *OGG1* and one in *MUTYH* (c.-127C>T) [31]. For the common *MUTYH* variant p.Gln338His, no significant difference was found between 547 BC patients and 287 controls [32]. Although our study reports the largest case–control study so far, the power was insufficient to detect frequency differences below a certain mutation frequency. We expect that an OR of 2.0 (incident BC patient group) or 2.6 (BRCAx group) or more has been excluded by our study, but for detection of smaller risks, larger studies are needed.

**Table 3:** Incident BC patient group (ORIGO) genotypes in relation to ductal, lobular or other histology types

Variant	Genotype (No.)	Ductal			Lobular			Other		
		No. (%)	OR (95% CI)	*p*	No. (%)	OR (95% CI)	*p*	No. (%)	OR (95% CI)	*p*
c.536A>G (p.Tyr179Cys)	AA (1,310)	1,136 (86.7)		*0.035*	179 (13.8)		0.152	67 (5.2)		1.000
	AG (10)	6 (60.0)	*0.23 (0.06–0.82)*		3 (30.0)	2.68 (0.69–10.45)		1 (10.0)	2.04 (0.25–16.32)	
c.925C>T (p.Arg309Cys)	CC (1,310)	1,135 (86.6)		*0.036*	179 (13.8)		*0.039*	67 (5.2)		0.276
	CT (6)	3 (50.0)	*0.15 (0.03–0.77)*		3 (50.0)	*6.25 (1.25–31.19)*		1 (16.7)	3.67 (0.42–31.85)	
c.1187G>A (p.Gly396Asp)	GG (1,307)	1,130 (86.5)		1.000	181 (14.0)		1.000	67 (5.2)		1.000
	GA (14)	12 (85.7)	0.94 (0.21–4.23)		2 (14.3)	1.03 (0.23–4.62)		1 (7.1)	1.41 (0.18–10.92)	
c.1214C>T (p.Pro405Leu)	CC (1,320)	1,141 (86.4)		1.000	183 (14.0)		1.000	68 (5.2)		1.000
	CT (2)	2 (100)	–		0	–		0	–	
Four variants with MAF <1% combined	Major (1,275)	1,107 (86.8)		*0.021*	173 (13.7)		0.074	65 (5.2)		0.408
	Minor (32)	23 (71.9)	*0.39 (0.18–0.85)*		8 (25.0)	2.09 (0.93–4.75)		3 (9.4)	1.90 (0.57–6.41)	
c.1544C>T (p.Ser515Phe)	CC (1,280)	1,111 (86.8)		*0.038*	171 (13.5)		*0.005*	64 (5.1)		0.261
	CT (40)	30 (75.0)	*0.46 (0.22–0.95)*		12 (30.8)	*2.85 (1.42–5.74)*		4 (10.3)	2.15 (0.74–6.23)	
c.37-2487T>G	GG (614)	530 (86.3)		0.952	82 (13.5)		0.403	36 (5.9)		0.710
	GT (580)	505 (87.1)	1.08 (0.76–1.49)		74 (12.8)	0.94 (0.67–1.32)		28 (4.8)	0.81 (0.49–1.34)	
	TT (155)	132 (85.2)	0.91 (0.55–1.50)		27 (17.6)	1.37 (0.86–2.21)		9 (5.9)	0.99 (0.47–2.10)	
c.64G>A (p.Val22Met)	GG (1,245)	1,072 (86.1)		0.552	176 (14.3)		0.635	68 (5.5)		0.361
	GA (131)	116 (88.5)	1.25 (0.71–2.19)		16 (12.3)	0.84 (0.49–1.46)		5 (3.8)	0.69 (0.27–1.73)	
	AA (7)	6 (85.7)	0.97 (0.12–8.09)		1 (14.3)	1.00 (0.12–8.36)		0	–	
c.504+35G>A	GG (1,133)	976 (86.1)		0.780	160 (14.3)		0.779	61 (5.4)		0.241
	GA (228)	205 (89.9)	1.43 (0.90–2.28)		26 (11.5)	0.78 (0.50–1.21)		9 (4.0)	0.72 (0.35–1.47)	
	AA (15)	10 (66.7)	*0.32 (0.11–0.95)*		6 (40.0)	*4.00 (1.41–11.40)*		0	–	
c.1014G>C (p.Gln338His)	GG (819)	706 (86.2)		0.747	111 (13.7)		0.242	44 (5.4)		1.000
	GC (466)	410 (88.0)	1.17 (0.83–1.65)		63 (13.6)	1.00 (0.72–1.39)		20 (4.3)	0.79 (0.46–1.36)	
	CC (74)	59 (79.7)	0.63 (0.35–1.15)		16 (22.2)	1.80 (1.00–3.26)		6 (8.2)	1.56 (0.64–3.80)	
Haplotypes of 5 variants										
1 (All major alleles)	GGGGC (1,622)	1,402 (86.4)		0.974	217 (13.4)		0.283	89 (5.5)		0.356
2 (37-2487**T**, 1014**C)**	**T**GG**C**C (562)	484 (86.1)		0.880	84 (14.9)		0.446	29 (5.2)		0.990
3 (37-2487**T**, 504 + 35**A**)	**T**G**A**GC (253)	218 (86.2)		0.898	37 (14.6)		0.726	11 (4.3)		0.499
4 (64A)	G**A**GGC (138)	124 (89.9)		0.241	16 (11.6)		0.438	3 (2.2)		0.100
5 (37-2487**T,** 1014**C**, 1544**T**)	**T**GG**CT** (39)	29 (74.4)		*0.029*	12 (30.8)		*0.002*	4 (10.3)		0.143

*p* values below 0.05 and odds ratios with CIs not including the value 1 are italicized. Numbers and percentages of patients with a histological type are given per genotype. In case of multiple tumors per patient with different histology, or multiple histological types within one tumor, each type was counted. To tests for associations between genotypes and tumor histology types, for each histology type (ductal, lobular, or other) a comparison was made between having this specific type or not. Totals may vary due to missing data and multiple possible histological types per patient

### Association of haplotypes

To investigate a potential effect of untyped variants in linkage disequilibrium (LD) with the genotyped *MUTYH* variants, an analysis of haplotypes was performed. One block with five haplotypes with a frequency above 1% was discerned from the unphased genotypes of the four tagging polymorphisms and one candidate variant. Two haplotypes had a frequency above 20% (Tables 2, 3). The fourth haplotype, carrying the minor allele of variant c.64G>A (p.Val22Met) was less prevalent in the incident BC patient (5.2%, *p* = 0.10) and BRCAx groups (5.1%, *p* = 0.20) in comparison to in the controls (6.5%). Conversely, the third haplotype, carrying the minor allele of variant c.504+35G>A, was more prevalent among incident BC patients (9.5%, *p* = 0.11) and BRCAx subjects (9.4%, *p* = 0.30) relative to controls (8.3%). We conclude that none of the investigated *MUTYH* haplotypes are associated with BC, excluding the role of unknown common variants in strong LD with the genotyped variants.

### Hereditary predisposition factors and tumor characteristics

Next, we evaluated a possible association of *MUTYH* variants with clinical features being suggestive of a hereditary predisposition. Therefore, the parameters age at diagnosis, multiple primary breast tumors in one patient (Supplementary Table S1) and BC family history (Supplementary Table S2) were analyzed in the incident BC group (ORIGO cohort). Also an association with CRC in the index patient and/or family history was tested (Supplementary Table S2). Heterozygous carriers of mutation, p.Tyr179Cys, were older at BC diagnosis (60.8 years, *n* = 11) compared to carriers of two wild-type alleles (52.7 years, *n* = 1,358, *p* = 0.01). Among heterozygous carriers of polymorphism, p.Ser515Phe, the frequency of patients with multiple primary breast tumors was higher (9/34, 21%) than in carriers of two wild-type alleles (149/1,177, 11%, *p* = 0.06). Analyses in larger groups are necessary to confirm these trends.

To look for possible specific *MUTYH* effects on breast tumor characteristics, we analyzed available immunohistochemistry data and tumor histology in the incident BC patients (ORIGO cohort) in relation to *MUTYH* genotypes. No statistically significant differences were found for ER and PR (Supplementary Table S3). Heterozygotes for minor alleles of several rare variants (p.Tyr179Cys, p.Arg309Cys, and p.Ser515Phe) showed a significantly higher frequency of lobular histology, combined with a lower frequency of ductal histology. For example, this effect was the strongest among p.Ser515Phe heterozygotes, of whom 12/40 (31%) had one or more breast tumors with lobular histology, compared to 171/1,280 (14%) with two wild-type, p.Ser515Phe, alleles (OR 2.9, *p* = 0.005). Also, the homozygous genotypes of the common variants, c.37-2487T>G, c.504+35G>A, and p.Gln338His, but not p.Val22Met, showed a higher frequency of lobular histology and lower frequency of ductal histology (Table 3). As we did not correct for multiple testing, we considered the significantly higher frequency of lobular histology as a trend towards association of *MUTYH* variants with breast tumor histology type. An association of *MUTYH* variants with BC histology has not yet been reported before. The one study investigating BC histology type in relation to *MUTYH* mutations reported no differences for ductal or other BC histology type between 30 patients heterozygous for p.Gly396Asp or p.Tyr179Cys and 359 patients homozygous wild-type for these mutations [29].

Associations with specific tumor characteristics have been found for high-risk and low risk BC genes. *BRCA1*-related breast tumors are often “triple-negative” (i.e., showing negative immunohistochemical staining for estrogen receptor (ER), progesterone receptor (PR), and HER2/neu) [33]. Also for low risk loci, associations with breast tumor subtypes have been described [33, 34]. Lobular and ductal BCs are believed to follow different pathways in tumorigenesis [35]. It might be interesting to look for associations of *MUTYH* variants, specifically in a large group of lobular BC patients.

## Conclusions

Our results do not confirm the data from two earlier studies which suggested a significantly increased BC risk among carriers of bi-allelic *MUTYH* mutations [4, 6]. No bi-allelic carriers of pathogenic *MUTYH* mutations were found in two large groups of BC patients. Also an effect of mono-allelic *MUTYH* mutations as earlier suggested could not be significantly confirmed [7, 29]. Although our study is the largest case–control study for *MUTYH* variants and BC to date, the power is expected to be insufficient for ORs below 2.0. Several trends towards association were observed between different *MUTYH* variants and clinical parameters, such as the BRCAx phenotype and lobular type of BC histology, which might be interesting for future studies.

## Electronic supplementary material

Below is the link to the electronic supplementary material.

Supplementary material 1 (DOC 197 kb)
