# Supplementary material for: MUTYH gene variants and breast cancer in a Dutch case–control study
Source: Breast Cancer Res Treat. 2012 Feb 2;134(1):219–27. doi: 10.1007/s10549-012-1965-0 (PMC3397219; doi:10.1007/s10549-012-1965-0)
Supplement: Supplementary file 1 — Supplementary material 1 (DOC 197 kb) [file 10549_2012_1965_MOESM1_ESM.doc]

SUPPLEMENTARY TABLES

Supplementary Table S1**:** Genotypes in relation to age at diagnosis and multiple primary tumors per patient in the incident BC patient group (ORIGO cohort).

| Variant | Geno-type | Age diagnosis in years | |  | Age groups in years, No. (%) | | | |  | Breast tumors, No. (%) | |  |  |
| --- | --- | --- | --- | --- | --- | --- | --- | --- | --- | --- | --- | --- | --- |
|  |  | N= | mean (range) | P | <40 | 40-49 | 50-59 | ≥60 | P | 1 | >1 | P | OR (95% CI) |
| c.536A>G | AA | 1358 | 52.7 (21-87) | **0.02** | 158 (11.6) | 383 (28.2) | 455 (33.5) | 362 (26.7) | **0.04** | 1199 (88.3) | 159 (11.7) | 0.38 |  |
| (p.Tyr179Cys) | AG | 11 | 60.8 (45-77) |  | 0 | 1 (9.1) | 5 (45.5) | 5 (45.5) |  | 11 (100.0) | 0 |  | - |
| c.925C>T | CC | 1359 | 52.8 (21-87) | 0.54 | 156 (11.5) | 382 (28.1) | 456 (33.6) | 365 (26.9) | 0.84 | 1201 (88.4) | 158 (11.6) | 0.62 |  |
| (p.Arg309Cys) | CT | 6 | 50.0 (36-60) |  | 1 (16.7) | 1 (16.7) | 3 (50.0) | 1 (16.7) |  | 6 (100.0) | 0 |  | - |
| c.1187G>A | GG | 1356 | 52.8 (21-87) | 0.39 | 157 (11.6) | 380 (28.0) | 453 (33.4) | 366 (27.0) | 0.28 | 1200 (88.5) | 156 (11.5) | 0.72 |  |
| (p.Gly396Asp) | GA | 14 | 55.4 (44-67) |  | 0 | 3 (21.4) | 7 (50.0) | 4 (28.6) |  | 13 (92.9) | 1 (7.1) |  | 0.59 (0.08-4.55) |
| c.1214C>T | CC | 1369 | 52.8 (21-87) | 0.33 | 157 (11.5) | 383 (28.0) | 461 (33.7) | 368 (26.9) | 0.73 | 1211 (88.5) | 158 (11.5) | 0.56 |  |
| (p.Pro405Leu) | CT | 2 | 45.0 (30-60) |  | 1 (50.0) | 0 | 0 | 1 (50.0) |  | 2 (100.0) | 0 |  | - |
| 4 variants ( MAF  <1%) combined | Major | 1323 | 52.7 (21-87) | 0.15 | 154 (11.6) | 376 (28.4) | 442 (33.4) | 351 (26.5) | 0.07 | 1167 (88.2) | 156 (11.8) | 0.17 |  |
|  | Minor | 33 | 55.6 (30-77) |  | 2 (6.1) | 5 (15.2) | 15 (45.5) | 11 (33.3) |  | 32 (97.0) | 1 (3.0) |  | 0.23 (0.03-1.72) |
| c.1544C>T | CC | 1326 | 52.9 (21-87) | 0.41 | 151 (11.4) | 370 (27.9) | 445 (33.6) | 360 (27.1) | 0.17 | 1177 (88.8) | 149 (11.2) | 0.06 |  |
| (p.Ser515Phe) | CT | 43 | 51.4 (31-86) |  | 7 (16.3) | 14 (32.6) | 13 (30.2) | 9 (20.9) |  | 34 (79.1) | 9 (20.9) |  | 2.09 (0.98-4.45) |
| c.37-2487T>G | GG | 635 | 53.0 (23-86) | 0.47 | 71 (11.2) | 182 (28.7) | 203 (32.0) | 179 (28.2) | 0.34 | 566 (89.1) | 69 (10.9) | 0.66 |  |
|  | GT | 603 | 52.6 (21-87) |  | 64 (10.6) | 177 (29.4) | 204 (33.8) | 158 (26.2) |  | 533 (88.4) | 70 (11.6) |  | 1.08 (0.76-1.53) |
|  | TT | 160 | 51.8 (26-86) |  | 26 (16.2) | 35 (21.9) | 64 (40.0) | 35 (21.9) |  | 141 (88.1) | 19 (11.9) |  | 1.11 (0.64-1.90) |
| c.64G>A | GG | 1291 | 52.7 (21-87) | 0.83 | 150 (11.6) | 361 (28.0) | 443 (34.3) | 337 (26.1) | 0.67 | 1140 (88.3) | 151 (11.7) | 0.81 |  |
| (p.Val22Met) | GA | 136 | 53.2 (29-82) |  | 14 (10.3) | 42 (30.9) | 40 (29.4) | 40 (29.4) |  | 122 (89.7) | 14 (10.3) |  | 0.87 (0.47-1.55) |
|  | AA | 7 | 54.3 (36-69) |  | 1 (14.3) | 2 (28.6) | 1 (14.3) | 3 (42.9) |  | 6 (85.7) | 1 (14.3) |  | 1.26 (0.15-10.52) |
| c.504+35G>A | GG | 1175 | 52.7 (21-87) | 0.99 | 137 (11.7) | 337 (28.7) | 387 (32.9) | 314 (26.7) | 0.70 | 1041 (88.6) | 134 (11.4) | 0.55 |  |
|  | GA | 236 | 52.6 (26-82) |  | 25 (10.6) | 64 (27.1) | 87 (36.9) | 60 (25.4) |  | 206 (87.3) | 30 (12.7) |  | 1.13 (0.74-1.73) |
|  | AA | 15 | 52.5 (38-69) |  | 1 (6.7) | 5 (33.3) | 5 (33.3) | 4 (26.7) |  | 13 (86.7) | 2 (13.3) |  | 1.20 (0.27-5.35) |
| c.1014G>C | GG | 846 | 53.0 (23-86) | 0.14 | 88 (10.4) | 243 (28.7) | 276 (32.6) | 239 (28.3) | **0.03** | 748 (88.4) | 98 (11.6) | 0.68 |  |
| (p.Gln338His) | GC | 485 | 52.2 (21-87) |  | 56 (11.5) | 146 (30.1) | 172 (35.5) | 111 (22.9) |  | 429 (88.5) | 56 (11.5) |  | 1.00 (0.70-1.41) |
|  | CC | 77 | 50.8 (26-78) |  | 16 (20.8) | 17 (22.1) | 26 (33.8) | 18 (23.4) |  | 66 (85.7) | 11 (14.3) |  | 1.27 (0.65-2.49) |

P-values below 0.05 are shown in bold. P-values for comparing age means were calculated by one way ANOVA.

OR: Odds ratio. CI: confidence interval.

Supplementary Table S2**:** Genotypes in relation to BC family history and CRC in index patient and/or family in the incident BC patient group (ORIGO cohort).

| Variant | Geno-type |  | Family history BC, No. (%) | | P | OR (95% CI) | CRC in index and/or family, No. (%) | | P | OR (95% CI) |
| --- | --- | --- | --- | --- | --- | --- | --- | --- | --- | --- |
|  |  | N= | None | ≥1 1st/2nd DGR |  |  | None | Index / ≥1 1st/2nd DGR |  |  |
| c.536A>G | AA | 1358 | 868 (63.9) | 490 (36.1) | 0.76 |  | 1140 (83.9) | 218 (16.1) | 0.40 |  |
| (p.Tyr179Cys) | AG | 11 | 8 (72.7) | 3 (27.3) |  | 0.66 (0.18-2.52) | 8 (72.7) | 3 (27.3) |  | 1.96 (0.52-7.45) |
| c.925C>T | CC | 1359 | 867 (63.8) | 492 (36.2) | 1.00 |  | 1139 (83.8) | 220 (16.2) | 1.00 |  |
| (p.Arg309Cys) | CT | 6 | 4 (66.7) | 2 (33.3) |  | 0.88 (0.16-4.83) | 5 (83.3) | 1 (16.7) |  | 1.04 (0.12-8.91) |
| c.1187G>A | GG | 1356 | 867 63.9) | 489 (36.1) | 0.60 |  | 1135 (83.7) | 221 (16.3) | 0.48 |  |
| (p.Gly396Asp) | GA | 14 | 10 (71.4) | 4 (28.6) |  | 0.71 (0.22-2.27) | 13 (92.9) | 1 (7.1) |  | 0.40 (0.05-3.04) |
| c.1214C>T | CC | 1369 | 877 (64.1) | 492 (35.9) | 1.00 |  | 1147 (83.8) | 222 (16.2) | 1.00 |  |
| (p.Pro405Leu) | CT | 2 | 1 (50.0) | 1 (50.0) |  | 1.78 (0.11-28.56) | 2 (100) | 0 |  | - |
| 4 variants (MAF  <1%) combined | Major | 1323 | 842 (63.6) | 481 (36.4) | 0.58 |  | 1108 (83.7) | 215 (16.3) | 1.00 |  |
|  | Minor | 33 | 23 (69.7) | 10 (30.3) |  | 0.76 (0.36-1.61) | 28 (84.8) | 5 (15.2) |  | 0.92 (0.35-2.41) |
| c.1544C>T | CC | 1326 | 845 (63.7) | 481 (36.3) | 0.20 |  | 1108 (83.6) | 218 (16.4) | 0.29 |  |
| (p.Ser515Phe) | CT | 43 | 32 (74.4) | 11 (25.6) |  | 0.60 (0.30-1.21) | 39 (90.7) | 4 (9.3) |  | 0.52 (0.18-1.47) |
| c.37-2487T>G | GG | 635 | 400 (63.0) | 235 (37.0) | 0.33 |  | 529 (83.3) | 106 (16.7) | 0.46 |  |
|  | GT | 603 | 388 (64.3) | 215 (35.7) |  | 0.94 (0.75-1.19) | 505 (83.7) | 98 (16.3) |  | 0.97 (0.72-1.31) |
|  | TT | 160 | 108 (67.5) | 52 (32.5) |  | 0.82 (0.57-1.18) | 139 (86.2) | 22 (13.8) |  | 0.80 (0.49-1.30) |
| c.64G>A | GG | 1291 | 829 (64.2) | 462 (35.8) | 0.70 |  | 1079 (83.6) | 212 (16.4) | 0.51 |  |
| (p.Val22Met) | GA | 136 | 88 (64.7) | 48 (35.3) |  | 0.98 (0.68-1.42) | 115 (84.6) | 21 (15.4) |  | 0.93 (0.57-1.51) |
|  | AA | 7 | 3 (42.9) | 4 (57.1) |  | 2.39 (0.53-10.74) | 7 (100) | 0 |  | - |
| c.504+35G>A | GG | 1175 | 752 (64.0) | 423 (36.0) | 0.70 |  | 981 (83.5) | 194 (16.5) | 0.74 |  |
|  | GA | 236 | 152 (64.4) | 84 (35.6) |  | 0.98 (0.73-1.32) | 203 (86.0) | 33 (14.0) |  | 0.82 (0.55-1.23) |
|  | AA | 15 | 11 (73.3) | 4 (26.7) |  | 0.46 (0.21-2.04) | 11 (73.3) | 4 (26.7) |  | 1.84 (0.58-5.84) |
| c.1014G>C | GG | 846 | 532 (62.9) | 314 (37.1) | 0.44 |  | 710 (83.9) | 136 (16.1) | 0.81 |  |
| (p.Gln338His) | GC | 485 | 322 (66.4) | 163 (33.6) |  | 0.86 (0.68-1.08) | 404 (83.3) | 81 (16.7) |  | 1.05 (0.78-1.41) |
|  | CC | 77 | 48 (62.3) | 29 (37.7) |  | 1.02 (0.63-1.66) | 67 (87.0) | 10 (13.0) |  | 0.78 (0.39-1.55) |

≥1 1st/2nd DGR: one or more first and/or second degree relative affected. OR: Odds ratio. CI: confidence interval.

**Supplementary Table S3: Genotypes in relation to hormone receptor status in the incident BC patient group (ORIGO cohort)**

| Variant | Geno-type | ER, No. (%) | | | P | PR, No. (%) | | | P |
| --- | --- | --- | --- | --- | --- | --- | --- | --- | --- |
|  |  | N= | neg | pos |  | N= | neg | pos |  |
| c.536A>G | AA | 1008 | 258 (25.6) | 750 (74.4) | 1.00 | 836 | 342 (40.9) | 494 (59.1) | 0.75 |
| (p.Tyr179Cys) | AG | 10 | 3 (30.0) | 7 (70.0) |  | 9 | 3 (33.3) | 6 (66.7) |  |
| c.925C>T | CC | 1015 | 260 (25.6) | 755 (74.4) | 1.00 | 842 | 345 (41.0) | 497 (59.0) | 0.65 |
| (p.Arg309Cys) | CT | 4 | 1 (25.0) | 3 (75.0) |  | 4 | 1 (25.0) | 3 (75.0) |  |
| c.1187G>A | GG | 1007 | 258 (25.6) | 749 (74.4) | 1.00 | 835 | 341 (40.8) | 494 (59.2) | 1.00 |
| (p.Gly396Asp) | GA | 12 | 3 (25.0) | 9 (75.0) |  | 10 | 4 (40.0) | 6 (60.0) |  |
| c.1214C>T | CC | 1019 | 261 (25.6) | 758 (74.4) | 0.26 | 844 | 345 (40.9) | 499 (59.1) | 0.41 |
| (p.Pro405Leu) | CT | 1 | 1 (100) | 0 |  | 1 | 1 (100) | 0 |  |
| 4 variants (MAF  <1%) combined | Major | 983 | 251 (25.5) | 732 (74.5) | 0.66 | 817 | 333 (40.8) | 484 (59.2) | 0.84 |
|  | Minor | 27 | 8 (29.6) | 19 (70.4) |  | 24 | 9 (37.5) | 15 (62.5) |  |
| c.1544C>T | CC | 990 | 254 (25.7) | 736 (74.3) | 0.68 | 816 | 332 (40.7) | 484 (59.3) | 0.45 |
| (p.Ser515Phe) | CT | 31 | 9 (29.0) | 22 (71.0) |  | 29 | 14 (48.3) | 15 (51.7) |  |
| c.37-2487T>G | GG | 488 | 126 (25.8) | 362 (74.2) | 1.00 | 400 | 165 (41.2) | 235 (58.8) | 0.88 |
|  | GT | 446 | 113 (25.3) | 333 (74.7) |  | 374 | 143 (38.2) | 231 (61.8) |  |
|  | TT | 114 | 30 (26.3) | 84 (73.7) |  | 95 | 41 (43.2) | 54 (56.8) |  |
| c.64G>A | GG | 968 | 242 (25.0) | 726 (75.0) | 0.26 | 807 | 325 (40.3) | 482 (59.7) | 0.44 |
| (p.Val22Met) | GA | 100 | 33 (33.0) | 67 (67.0) |  | 78 | 34 (43.6) | 44 (56.4) |  |
|  | AA | 4 | 0 | 4 (100) |  | 3 | 2 (66.7) | 1 (33.3) |  |
| c.504+35G>A | GG | 884 | 222 (25.1) | 662 (74.9) | 0.49 | 733 | 299 (40.8) | 434 (59.2) | 1.00 |
|  | GA | 174 | 49 (28.2) | 125 (71.8) |  | 146 | 57 (39.0) | 89 (61.0) |  |
|  | AA | 8 | 2 (25.0) | 6 (75.0) |  | 7 | 4 (57.1) | 3 (42.9) |  |
| c.1014G>C | GG | 643 | 172 (26.7) | 471 (73.3) | 0.34 | 527 | 216 (41.0) | 311 (59.0) | 0.61 |
| (p.Gln338His) | GC | 358 | 86 (24.0) | 272 (76.0) |  | 302 | 119 (39.4) | 183 (60.6) |  |
|  | CC | 55 | 13 (23.6) | 42 (76.4) |  | 47 | 18 (38.3) | 29 (61.7) |  |
